# The Sinus Tract in Bone and Joint Infection: Minimally Invasive Salvation or Prolonged Suffering? A Multicenter Study

**DOI:** 10.3390/jpm13050737

**Published:** 2023-04-26

**Authors:** Sebastian Martin Klim, Florian Amerstorfer, Martin A. McNally, Rihard Trebse, Urban Slokar, Irene Katharina Sigmund, Andrzej Hecker, Patrick Reinbacher, Lukas Leitner, Gerwin Alexander Bernhardt, Andreas Leithner, Sophie Wanko, Mathias Glehr

**Affiliations:** 1 Department of Orthopaedics and Trauma, Medical University of Graz, 8036 Graz, Austria; sebastian.klim@medunigraz.at (S.M.K.);; 2 The Bone Infection Unit, Oxford University Hospitals, Oxford OX3 7HE, UK; 3 Orthopaedic Hospital Valdoltra, 6280 Ankaran, Slovenia; 4 Department of Orthopaedics and Tramatology, Medical University of Vienna, Spitalgasse 23, 1090 Vienna, Austria; 5 Division of Plastic, Aesthetic and Reconstructive Surgery, Department of Surgery, Medical University of Graz, 8036 Graz, Austria

**Keywords:** osteomyelitis, periprosthetic joint infection, salvage procedure, sinus tract, quality of life

## Abstract

This study assessed the quality of life (QOL) and the functional outcome in daily living in patients with a chronic, treatment-resistant periprosthetic joint infection (PJI) or osteomyelitis, living with a natural or iatrogenic sinus tract. Methods: A follow-up examination in three national reference centers for septic bone and joint surgery was performed utilizing the Hospital Anxiety and Depression Scale (HADS-D/A), the Visual Analogue Scale (VAS), and the Short Form-36 (SF-36) score, including patients with a chronic sinus tract due to treatment-resistant PJI or osteomyelitis. Results: In total, 48 patients were included, with a mean follow-up time of 43.1 ± 23.9 months. The mean SF-36 Mental Component Summary (MCS) was 50.2 (±12.3) and the Physical Component Summary (PCS) was 33.9 (±11.3). The mean HADS-D was 6.6 (±4.4) and HADS-A was 6.2 (±4.6), and the VAS was 3.4 (±2.6). The SF-36 MCS showed no significant differences between the study group and the standard population (47.0, *p* = 0.10), as well as the HADS-A. The PCS in the study population was significantly worse (50.0, *p* < 0.001), as was the HADS-D. Conclusions: A sinus tract represents a treatment option in selected cases with an acceptable QOL. The treatment should be considered for multimorbid patients with a high perioperative risk or if the bone or soft tissue quality prevents surgery.

## 1. Introduction

In recent years, major advances have been achieved in the treatment of chronic musculoskeletal infections. The cure rates of periprosthetic joint infections (PJI) have risen to over 90% for one- and two-stage exchange [1,2,3]. Further, improved surgical techniques and local high-dose antibiotic treatment are powerful tools in the treatment of osteomyelitis, leading to cure rates from 79% to 97% in the literature [4,5]. However, in PJI, as well as in osteomyelitis, cases remain in which the infection cannot be cured and must be accepted as treatment-resistant. Due to the increased life expectancy, increasing co-morbidities in the elderly, and constantly rising numbers in primary total hip and knee arthroplasty, cases of failed PJI or osteomyelitis treatment are becoming more frequent [6,7,8]. In these cases, further surgical treatment may be contraindicated due to the risks of severe comorbidities. Additionally, the prospects of success may be low, due to compromised bone stock and/or lack of soft tissue coverage, often following multiple revision surgeries. In this situation, there are few surgical options, all of which are associated with either severe loss of function or quality of life (QOL). Girdlestone resection arthroplasty, amputation, or arthrodesis may not be acceptable in many patients [9,10,11].

Maintaining a sinus tract (either spontaneous or iatrogenic) represents a different therapy approach as a minimally invasive treatment option. The minimally invasive nature of the iatrogenic sinus tract creation allows a broad range of indications in patients with severe comorbidities. Our hypothesis was that the minimal invasive sinus tract treatment can achieve the salvage of the limb with considerable function. The drawback to this approach is the assumed decrease in QOL of patients with a pus-producing sinus tract, which necessitates constant wound dressings and certain hygiene measures. In addition, chronic pain and the possibility of a spontaneous closure of the sinus tract with the need for surgical re-opening must be considered [12]. Very little evidence is available investigating these suspected drawbacks and the quality of life of patients living with a chronic sinus tract to date. The objective of the present study was to assess the quality of life and the functional outcome in daily living in patients with a chronic, treatment-resistant PJI or osteomyelitis living with a natural or iatrogenic sinus tract.

## 2. Materials and Methods

This study was performed in line with the principles of the Declaration of Helsinki. Approval was granted by the institutional Ethics Committee (28–210 ex 15/16) of the Medical University of Graz (Graz, Austria). The methods were carried out in accordance with relevant guidelines and regulations. Informed consent was obtained from all individual participants included in the study.

### 2.1. Patient Selection

Patients were recruited with a chronic sinus tract (existing for two months or more) due to treatment-resistant PJI or osteomyelitis cases in three international reference centers for septic bone and joint surgery. Two centers performed a retrospective (Department of Orthopaedics and Trauma, Medical University of Graz, Austria; Orthopaedic Hospital Valdoltra, Ankaran, Slovenia) patient recruitment, while one center performed prospective (Bone Infection Unit, Oxford University Hospitals, Oxford, England) recruitment. In all these cases, patients refused further extensive surgical treatment, or the attending surgeons decided that further curative interventions were either technically not feasible, the chance of infection eradication was too low, or it was too risky due to the patients’ comorbidities. A comprehensive follow-up examination at a single time point was performed, including the serum inflammatory markers leukocyte count (G/L, threshold 11.3 G/L) and C-reactive protein (CRP; mg/L, threshold 5 mg/L), the Cierny–Mader Host classification (osteomyelitis cases), and the McPherson Host classification (PJI cases) [13,14].

### 2.2. Study Assessment

To investigate the daily quality of life, the Hospital Anxiety and Depression Scale ranging from 0 (best) to 21 (worst) (HADS-A and HADS-D), the Visual Analogue Scale ranging from 0 (best) to 10 (worst) (VAS), and the Short Form-36 (SF-36) score were conducted (SF-36 MCS = Short Form-36 health survey mental component summary, SF-36 PCS = Short Form-36 health survey physical component summary, PF = physical functioning, RP = physical role functioning, BP = bodily pain, GH = general health perceptions, VT = vitality, SF = social role functioning, RE = emotional role functioning, MH = mental health) at the follow-up examination. Compared with other questionnaires designed to evaluate quality of life, the frequently used SF-36 questionnaire is short and flexible, which makes it much easier to administer. To compare and contextualize the results of the study group, the SF-36 score results were compared to those of the SF-36 reference dataset, representing the mean values of the general population. Additionally, the Oxford Hip or Knee score was completed in PJI cases after hip or knee arthroplasty, respectively, ranging from 12 (best) to 60 (worst). Using the hospital database and medical records, data were gathered on causal microorganisms, antibiotic suppression therapy, and necessary surgical interventions regarding the sinus tract (differentiating between sinus tract re-opening in an operating room and standard sinus tract care in the outpatient department).

### 2.3. Iatrogenic Sinus Tract Treatment Technique

The most important element of successful sinus tract therapy is patient selection. First, curative surgery to treat osteomyelitis or PJI is not technically possible or feasible due to severe comorbidities. Second, alternative treatment, such as amputation and/or long-term antibiotic suppression, is not possible or is rejected by the patient. Equally indispensable is the comprehensive education of the patient about the necessary sinus tract care. This includes keeping the sinus tract open and regular dressing changes.

**Figure 1:**
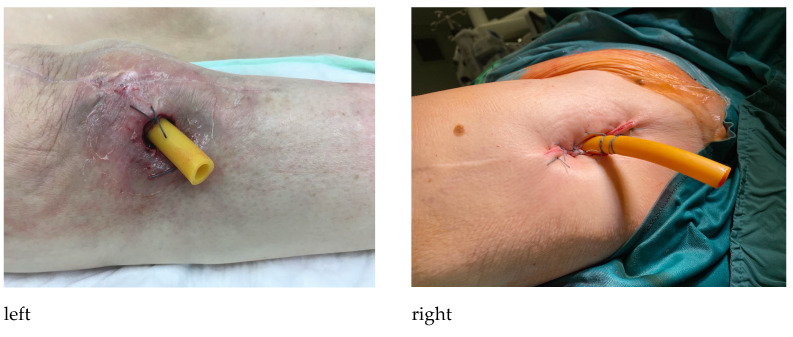
Surgical sinus tract creation using a large-diameter drainage tube and secured with non-absorbable suture material (**left**: right knee joint, **right**: left hip joint).

**Figure 2:**
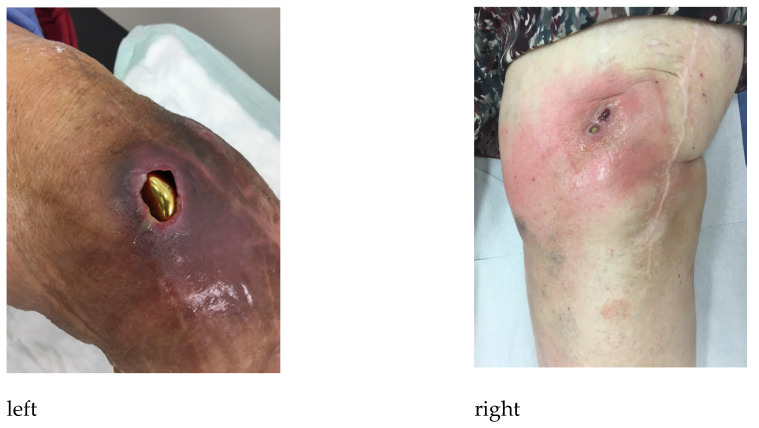
Naturally occurred sinus tract with visible prosthesis (**left**) and without visible prosthesis (**right**), following treatment-resistant PJI after total knee arthroplasty.

If the decision for sinus tract therapy was made and a natural sinus tract did not exist, an iatrogenic sinus tract creation was performed. This was carried out under strictly sterile conditions and will be explained using the example of a treatment-resistant PJI following total knee arthroplasty. The procedure was performed according to our in-house technique and should serve as an example. An adapted approach may be necessary depending on the individual characteristics of the patient. After sterile washing and draping of the limb, a local anesthetic was applied. Then, a circular skin excision of approximately 1–1.5 cm in diameter was made above and lateral to the patella. The knee joint was opened so that there is visual contact with the prosthesis. After hemostasis, the synovium was sutured to the cutis to prevent sinus tract closure. A large-diameter drainage tube was then inserted into the recessus suprapatellaris and secured with non-absorbable suture material (Figure 1). This drainage tube was left in situ for about 6 weeks until the fistula was stabilized. Figure 2 shows a naturally occurred sinus tract following treatment of PJI after total knee arthroplasty.

### 2.4. Statistical Analysis

Statistical analysis was performed using IBM SPSS Statistics (Version 27.0). We evaluated data with respect to parametric or non-parametric distribution using a Kolmogorov–Smirnov test where appropriate. To detect significant differences, we used the paired and unpaired t test. If parametric distribution was not found, the Mann–Whitney U test was performed. An analysis of variance (ANOVA) was performed to detect group differences regarding their means. A *p*-value of less than 0.05 was considered statistically significant.

## 3. Results

In total, 48 patients were included in the final analysis (23 patients were recruited prospectively and 25 retrospectively). Patient characteristics were as depicted in Table 1. The mean follow-up time was 43.1 months (SD ± 23.9, range 2–261 months) following iatrogenic or natural sinus tract formation. During this time, the number of surgical sinus tract re-openings at the outpatient department ranged from zero (*n* = 40), two to ten times (*n* = 3), and ten or more (*n* = 3). The number of necessary surgical sinus tract re-openings in the operating room ranged from zero (*n* = 40), one (*n* = 6), and two (*n* = 1), to eleven (*n* = 1). Six patients (12.5%) were treated with a permanent suppressive antibiotic therapy. At final follow-up, mean leucocyte count was 7.5 ± 1.9 (G/L) and CRP was 16.5 ± 22.3 (mg/L).

### 3.1. Daily Quality of Life

Basic score results were as depicted in Table 2. Female patients (w) had significantly lower PCS scores compared to male patients (m) (28.5 (w) vs. 36.6 (m), *p* = 0.017), while the MCS score was not significantly different (52.6 (w) vs. 49.0 (m), *p* = 0.33). While a higher BMI had no influence on the SF-36 QOL results, older patients had significantly lower PCS scores (*p* = 0.005) and higher MCS scores (*p* = 0.025). We report no significant differences in the SF-36 score results when the cause of the sinus tract (natural vs. surgical) and the number of surgical sinus tract re-openings were considered. When comparing the SF-36 score regarding the underlying pathology (osteomyelitis vs. PJI), we found a trend towards a higher PCS score in the osteomyelitis group, without statistical significance (31.1 vs. 36.9, *p* = 0.081), while the MCS score was significantly higher in the PJI group (54.6 vs. 45.4, *p* = 0.009).

While the SF-36 MCS score showed no significant differences between the study group and the standard population (50.1 vs. 47.0, *p* = 0.10), the PCS score in the study population was significantly worse (33.9 vs. 50.0, *p* < 0.001). For a comparative analysis between the HADS-D and SF-36 scores, we divided the study population into 3 subgroups, as proposed by the score guidelines, considering the respective HADS-D results (0–7 normal, 8–10 suspicious, >10 abnormal). The findings have shown significantly worse results for the abnormal group regarding the SF-36 subscales of bodily pain (63.1 vs. 34.0, *p* = 0.001), general health perceptions (62.6 vs. 43.8, *p* = 0.003), vitality (56.3 vs. 33.1, *p* = 0.001), and mental health (82.3 vs. 46.5, *p* < 0.001), compared to the group with normal HADS-D results.

### 3.2. Microorganisms

Twenty-one different microorganisms were found, with *Staphylococcus aureus* (18/45, 40%) and *Staphylococcus epidermidis* (8/45, 17.7%) being the most frequent. In three cases (3/48, 6.2%), no microorganisms could be isolated, and in thirteen cases (13/45, 28.9%), more than one microorganism was found. We found no statistically significant effect of the different microorganisms on the quality of life of the patients.

## 4. Discussion

The most important finding was the acceptable SF-36 MCS, HADS, and VAS scores, indicating a satisfactory mental health status and, ultimately, QOL of the patients, while the physical health was reduced (SF-36 PCS). This was not affected by the frequency of surgical sinus tract re-openings or the cause of the sinus tract. To our knowledge, the present study is the first describing the quality of life of sinus tract patients due to treatment-resistant chronic PJI and osteomyelitis, reporting valuable information on this therapy concept. Due to the infrequency of chronic sinus tract therapy and considering the exclusion of cases with a sinus tract duration below two months, the size of the study population is a strength of this study.

Due to the absence of a comparison group, we compared the SF-36 and the HADS results of our study population to the respective national reference values of the standard population [15,16]. As expected, the physical scores were lower, but it was encouraging that a persisting sinus tract did not create significant mental impairment. We deem adequate patient education and guidance before and during sinus tract therapy responsible for this. Particular attention should be paid to the necessary support in hygienic fistula care and information about alternative salvage procedures and their advantages and disadvantages. In our experience, this increases the resilience of patients against the undisputed drawbacks of chronic sinus tract therapy and directs the focus to the advantages. Troendlin et al. published first evidence regarding the quality of life of chronic sinus tract patients, reporting poor SF-36 results in 27 cases [12]. A recent study on the long-term QOL after successful TKA-PJI treatment reported slightly worse results, with a SF-36 PCS score of 24.82 ± 10.0 and a MCS score of 46.16 ± 13.3 at a follow-up of 4.9 ± 3.5 years [17]. Another study on this topic reported rather similar results to the present paper, with a SF-12 PCS score of 36.2 ± 11.3 and a MCS score of 52 ± 13.6 [18]. Although these studies included successfully and non-successfully treated PJI cases, both did not report better SF-36-based QOL results compared to the present study. Further, Helwig et al. found no significant QOL difference between patients with and without successful infection therapy [18]. Hotchen et al. reported a significantly reduced QoL with increasing complexity of the osteomyelitis according to the BACH classification regarding the EQ-5D (0.527 vs. 0.401, *p* < 0.05) and the VAS score. Patients not fit for surgery (due to co-morbidities) had similar score results compared to complex osteomyelitis cases [19].

In these heterogenous and difficult-to-treat cases, various treatment options are currently performed without a gold standard available [9]. To achieve limb preservation in PJI cases, the available surgical treatment alternatives range from a debridement and implant retention (DAIR) to a one-, two-, or multiple-stage exchange of the prosthesis, or an arthrodesis. However, even if the high perioperative risk of the multimorbid patient is left aside, the success rate (infection eradication and function) of these procedures is often lower than assumed, and it decreases with each additional revision procedure [7,10,20,21]. Sole antibiotic suppression therapy presents a possible nonsurgical treatment option for a few selected cases with certain causative microorganisms [22]. However, a recent study reported the development of antibiotic resistance in 23.1% of cases [23]. Above-knee amputation and hip disarticulation are drastic surgical treatment options for treatment-resistant bone and joint infections. They are often associated with poor functional outcomes, especially in cases following hip disarticulation [24,25,26]. Further, we regularly observe that patients are rather reluctant to agree to amputation and wish to exhaust limb-preserving options first.

In osteomyelitis cases, precise debridement and adequate dead space management are key, ideally combining the expertise of a specialized surgeon and an infectious disease specialist in a multidisciplinary approach [27,28,29]. To solve the problems of a persistent bacterial biofilm and dead space after surgical debridement, various substances have been developed to provide osteogenicity, osteoinductivity, and osteoconductivity, and to release high local doses of antibiotics. Studies have reported high cure rates of over 90% when these methods were adequately used [4,30]. However, despite these effective treatments, a minority of treatment-resistant cases remain. In some of these cases, patients will find the presence of a draining sinus unacceptable, and this is often the reason they seek medical treatment. However, our study shows that in those who accept the sinus, the mental and functional scores are acceptable.

## 5. Limitations

This study has several limitations that must be considered. First, the study population was rather heterogenic. We decided to include cases with different sinus tract locations and causes from three international reference centers to obtain robust data in an acceptable cohort size, which is representative of the types of cases commonly seen across Europe. Since the aim of the study was to investigate the QOL of patients living with a chronic sinus tract, we decided to include both PJI and osteomyelitis cases in this trial. Six patients received permanent antibiotic suppression therapy. This decision was made by the treating physician together with the infectious disease specialist in view of the germ spectrum, the inflammation parameters, and the immune status of the patients. Since two salvage procedures were combined here, this needs to be mentioned as a limitation with regard to the homogeneity of the study group. Second, the present study was designed without a comparison group (e.g., amputation), thus limiting the power of treatment recommendations derived from the presented data. We tried to mitigate this weakness by comparison with data from the standard population. Further, based on the small sample size, statistics must be interpreted with caution. Multiple testing might have overinterpreted our results; however, based on the small sample size, corrections such as the Bonferroni correction did not seem appropriate.

## 6. Conclusions

The presented data imply that a chronic sinus tract is a functionally acceptable treatment option in selected cases of treatment-resistant PJI or osteomyelitis, with reasonable quality of life but considerable drawbacks in physical function. The treatment should be considered for multimorbid patients in whom a major surgical intervention is associated with a major risk to life, or if the local bone or soft tissue status prevents successful surgical interventions. Patient selection and involvement, however, is key in the decision to maintain or create a permanent sinus.

## Figures and Tables

**Table 1:** Characteristics of the study population. Standard deviation (SD), body mass index (BMI), and periprosthetic joint infection (PJI). Cierny–Mader Host classification was used for osteomyelitis cases (not available in three cases) and McPherson Host classification for PJI cases.

Total, *N* = 48		McPherson Host Classification (*n* = 25)		Cierny–Mader Host Classification (*n* = 23)	
Age (years), mean (range)	63.6 (22.5–92.5)	IA1	*n* = 1	3A	*n* = 1
Female, *n* (%)	16 (33%)	IA2	*n* = 2	3B	*n* = 2
Right side, *n* (%)	22 (46%)	IA3	*n* = 1	3Bl	*n* = 9
BMI, mean (SD)	27.4 (±4.9)	IB2	*n* = 2	3Bls	*n* = 6
PJI/osteomyelitis cases, *n* (%)	25 (52%)/23 (48%)	IIIA1	*n* = 1	3Bs	*n* = 1
Cause of sinus tract (surgical/natural), *n* (%)	7 (15%)/41 (85%)	IIIA2	*n* = 5	4Bls	*n*= 1
Localization of the sinus tract, *n* (%)		IIIA3	*n* = 1		
Hip joint	10 (20%)	IIIB1	*n* = 1		
Knee joint	14 (29%)	IIIB2	*n* = 6		
Shoulder Joint	2 (4%)	IIIB3	*n* = 3		
Femur	7 (15%)	IIIC2	*n* = 2		
Tibia	11 (23%)				
Foot	1 (2%)				
Forearm	1 (2%)				
Humerus	2 (4%)				

**Table 2:** Mean and standard deviation (SD) of scores and SF-36 subscales (ranging from 0 to 100). SF-36 MCS = SF-36 mental component summary, SF-36 PCS = physical component summary, HADS-D = Hospital Anxiety and Depression Scale Depression Score, HADS-A = Hospital Anxiety and Depression Scale Anxiety Score, VAS = Visual Analogue Scale, PF = physical functioning, RP = physical role functioning, BP = bodily pain, GH = general health perceptions, VT = vitality, SF = social role functioning, RE = emotional role functioning, MH = mental health.

Scores	Scores at Follow-Up, Mean (SD)	SF-36 Subscales	SF-36 Subscales at Follow-Up Mean (SD)
SF-36 MCS	50.2 (±12.3)	PF	43.3 (±32.3)
SF-36 PCS	33.9 (±11.3)	RP	29.6 (±22.8)
HADS-D	6.6 (±4.4)	BP	51.1 (±28.5)
HADS-A	6.2 (±4.6)	GH	52.9 (±20.9)
VAS	3.4 (±2.6)	VT	44.8 (±23.1)
Oxford Hip Score	21.3 (±5.3)	SF	61.7 (±20.9)
Oxford Knee Score	15.6 (±7.3)	RE	47.1 (±29.7)
		MH	68.2 (±25.3)

## Data Availability

Detailed data supporting the results are available from the authors.
